# Comparative study between the roles of intrauterine misoprostol versus the sublingual route for prevention of postpartum blood loss in elective cesarean sections: a randomized controlled trial

**DOI:** 10.1186/s12884-024-06889-y

**Published:** 2024-10-29

**Authors:** Adel Atef, Hadeer Salah Eldin Abdelrahman Mohamed Shehata, Yasmin Ahmed Bassiouny, Hesham Gaber Al-Inany

**Affiliations:** 1 Ob/gyn Department, Faculty of Medicine, Cairo University, Cairo University, Cairo, Egypt; 2 Resident of obstetrics and gynecology, Helwan General Hospital, Cairo, Egypt

**Keywords:** Uterotonic, Ecbolic, Misoprostol, Sublingual, Intrauterine, Postpartum hemorrhage, Cesarean delivery

## Abstract

**Background:**

The prostaglandin E1 analog “misoprostol” is a drug that has powerful ecbolic effects and can be beneficial in the prevention and treatment of postpartum hemorrhage, which is the leading cause of maternal mortality worldwide.

**Objectives:**

To assess the value of adding intrauterine misoprostol together with intravenous oxytocin injection compared with sublingual misoprostol together with intravenous oxytocin injection during elective cesarean section to reduce blood loss intraoperatively and prevent postpartum hemorrhage.

**Methods:**

A total of 192 pregnant women were counseled and recruited from the labor and delivery unit at Kasr Al Aini Hospital, Cairo University, and equally randomized into two groups. Group (A) included 96 women who received intrauterine misoprostol (400 mg) + oxytocin. Group (B) included 96 women who received sublingual misoprostol (400 mg) + oxytocin. The primary outcome of our study was estimation of the amount of blood loss during and after cesarean delivery. The secondary outcomes were the incidence of PPH within the first 6 h after labor, the need for blood transfusion, the need for any supplementary ecbolic drugs, the need for additional surgical intervention for PPH, changes in hematocrit and hemoglobin in both groups after delivery, and the incidence of side effects of the study medications.

**Results:**

We observed a significant discrepancy between the two groups in terms of postoperative Hb and Hct, postoperative differences (pre- and post-Hb and post-Hct) and EBL favoring the intrauterine group. However, no significant difference was observed between the groups with respect to excessive blood loss > 1000 ml in the 1st six hours, the need for supplementary ecbolics, the necessity for blood or blood prod, the need for additional surgical intervention (for PPH) or side effects.

**Conclusion:**

Intrauterine misoprostol combined with oxytocin intravenous infusion is more effective than sublingual misoprostol combined with oxytocin intravenous infusion in lowering intraoperative blood loss and preventing postpartum hemorrhage in elective cesarean section.

**Trial registration:**

This trial was retrospectively registered with the ClinicalTrials.gov Registry on 12-April-2024 (registration number: NCT06364098).

**Supplementary Information:**

The online version contains supplementary material available at 10.1186/s12884-024-06889-y.

## Background

Globally, postpartum hemorrhage (PPH) is considered a major contributor to maternal morbidity and death, with an incidence ranging from 6 to 10.8%. It is thought to be the direct cause of 25% of all female mortalities [1]. Uterine atony, which occurs in both vaginal and cesarean births, is the most avoidable cause of PPH. Cesarean sections are becoming more common in both developed and underdeveloped countries [2]. The estimated amount of blood loss during postpartum hemorrhage varies depending on the mode of birth, with an average of 500 ml of blood or more during vaginal birth, 1000 ml or more during cesarean section (CS), and an average of 3500 ml during emergency hysterectomy; thus, preventing postpartum hemorrhage is critical for maternal safety [3].

During cesarean birth, oxytocin is commonly given to avoid uterine atony and excessive uterine hemorrhage. Despite their efficacy,  extra uterotonic medications are required in 10–40% of cases to accomplish enough uterine contractions [4].

The most commonly used supplementary uterotonic medication is a prostaglandin E1 analog (misoprostol), which specifically attaches to prostanoid receptors, promoting strong uterine contraction at therapeutic doses with few side effects [5]. For this reason, misoprostol can be an effective alternative to other conventional ecbolic medications. Moreover, it can be administered orally, sublingually, buccally, vaginally, rectally, or intrauterine. It is thought to be appropriate for global utilization, including low-resource settings in developing countries. This is attributable to its convenience for administration, thermal stability, reasonable price and availability [6]. Furthermore, pharmacokinetic studies demonstrated that it is better absorbed when taken sublingually than if it is administered orally or vaginally. As a result, sublingual administration for PPH prevention has been investigated and proven to be effective [7].

The pharmacological activity of misoprostol on uterine muscle fibers involves powerful myometrial contractility starting at the fundus near the cornua and progressing throughout the uterine body, compressing uterine vessels and reducing blood loss. The intrauterine use of misoprostol is closer to the targeted organ, which acts as an autocoid substance with a stronger impact [8]. As a result, the intrauterine route may be more effective than alternative routes.

In our study, we aimed to evaluate the effectiveness of combining the use of intrauterine misoprostol with oxytocin intravenous infusion rather than sublingual misoprostol combined with oxytocin intravenous infusion to reduce intraoperative blood loss and prevent postpartum hemorrhage in elective cesarean section.

## Participants and methods

### Study design

A prospective randomized control trial was carried out at the labor and delivery unit at Kasr Al Aini Hospital, Cairo University from January 2022 until February 2023.The clinical trial registration number is (NCT06364098), and the date of registration was at 12-April-2024. The current study followed the Consolidated Standards of Reporting Trials (CONSORT) guidelines.

### Study population

**Fig. 1:**
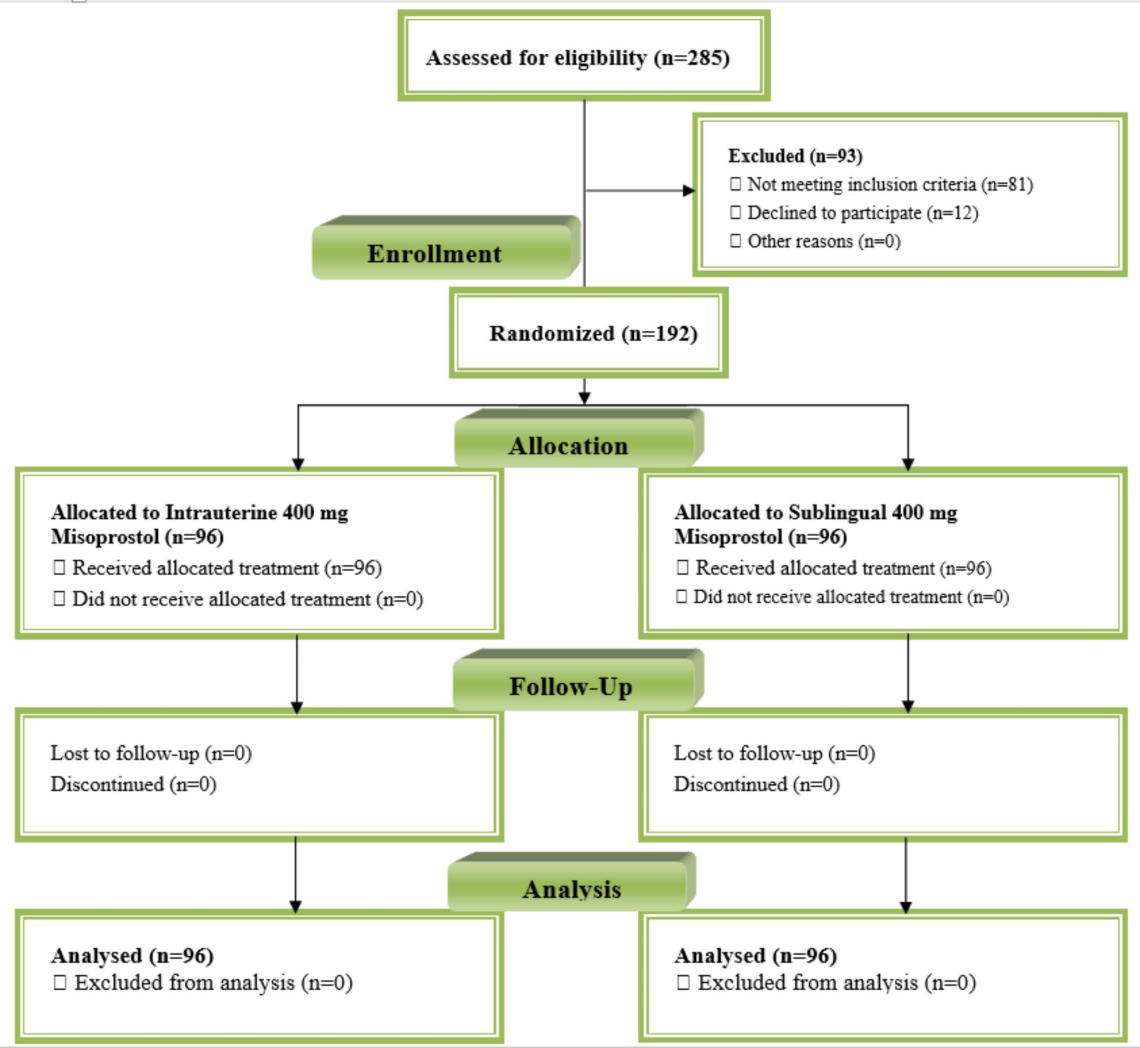
CONSORT Flow Diagram

The inclusion criteria for pregnant women were as follows: aged between 20 and 35 years, with a BMI < 30 kg/m2 and presenting with a full-term singleton healthy living fetus (gestational age > 39 weeks confirmed by the first day of the last menstrual period or first-trimester ultrasound scan) and being a candidate for elective cesarean delivery (previous one or two cesarean sections). The exclusion criteria for patients were as follows: uterine distension due to multiple pregnancies or polyhydramnios; multiparity (parity ≥ 3); uterine fibroids; antepartum hemorrhage presentation such as placenta previa, placental abruption or vasa previa; moderate to severe anemia with a hemoglobin level < 9 mg/dl; anticoagulant use during pregnancy; coagulopathy or thrombocytopenia or blood dyscrasias; hypertension; cardiovascular, DM, hepatic, or renal disorders; and any contraindication for the use of misoprostol or oxytocin as an allergy to prostaglandin and concomitant drugs that have drug interactions with prostaglandins such as topical dinoprostones or antacids containing magnesium. Figure 1 depicts patients’ dispositions.

### Study measurements

After receiving the research approval of the Scientific and Ethical Committee of the Obstetrics and Gynecology Department, Cairo University (IRB: MS-71-22), the study purpose was clarified simply and in a lay Arabic speech to all the women before they were registered in the study, and the enrolled women signed an informed consent form. Pregnant women who met all the inclusion criteria were evaluated for enrollment. Prior to the study, randomization schedules created by computer programs were designed and set in serially numbered secured opaque envelopes. Simple randomization was applied at a one-to-one ratio within both groups. After providing consent for participation, the recruited women opened sealed randomization envelopes to reveal the allocation. Participants were assigned sequential numbers (1–192) based on the time of admission. There were 96 patients in each group, and simple randomization (1:1) was utilized to allocate participants to the groups A and B. Utilizing MedCalc © version 13, a random number sequence was produced .

All participants were subjected to.

- Comprehensive medical history, including obstetric, medical, and surgical history, was obtained.
- A general examination excluded the presence of any abnormalities.
- Systematic obstetrical examinations.
- A transabdominal ultrasound was used to confirm the diagnosis and evaluation of the fetal condition.
- A blood sample was collected for complete blood counting, coagulation studies, and liver and kidney function tests.

All women were subjected to elective lower segment cesarean section by the same team under spinal anesthesia and antibiotic prophylaxis according to the hospital protocol.

The women were divided into two equal groups:

- Group A included 96 women who received 400 mcg of intrauterine misoprostol + IV oxytocin.
- Group B included 96 women receiving 400 mcg sublingual misoprostol + IV oxytocin.

Both groups were parallelly subjected to the same preparation. All cesarean deliveries were performed by a senior obstetrics and gynecology resident, and the elective sections were performed at 39th week of gestation via the following operative steps: transverse skin incision (Pfannenstiel), smiley incision at the lower uterine segment, immediate clamping of the cord within 30 seconds, placental extraction after spontaneous separation, two-layer closure of the uterine incision, and abdominal closure anatomically in layers. All surgical steps were performed with adequate hemostasis. Furthermore, we excluded sections exceeding 90 min from skin incision to skin closure.

After fetal head extraction, the participants in both groups were given 5 IU oxytocin (Syntocinon, Novartis, Basel, Switzerland) as an intravenous bolus, and the infusion of 10 IU oxytocin in 500 mL of Ringer lactate at a rate of 125 mL/hour was continued.

- For all women in group A, the surgeon placed two intrauterine misoprostol tablets (Misotac^®^ 200 micrograms, Sigma Ph., Egypt) at the fundus after placental delivery and swabbed the cavity followed by suturing the first layer of the uterus, whereas women in group B were given two sublingual misoprostol tablets (Misotac^®^ 200 micrograms, Sigma Ph., Egypt) after placental delivery.
- After uterine incision, nearly all liquor was collected in a separate suction tube and after delivery of the baby blood was collected in other suction tube and with the use of surgical towels that was weighted before and after the operation. Blood collection drapes were used during the first 6 h post operatively to collect the blood lost during this period. The amount of blood loss was determined through the standardized visual estimation method and rectified by measuring the amount of intra-operative blood loss and 6 h after surgery. The estimated total blood loss during cesarean section was measured after adding the volume of blood in the suction bottle to the soaked surgical towels (weighting method◊ 1 gram difference equal 1 ml of blood) then added to the amount of blood lost after section calculated by using blood collection drape [9–11]. Moreover, hemoglobin (HB) level and hematocrit value were repeated 24 h postpartum. The patient’s vitals were observed and recorded intra-operatively and postoperatively. The estimated blood loss was then calculated by multiplying estimated blood volume by the difference between preoperative and post operative hematocrit and all divided by the preoperative hematocrit as simplified by the following equation: EBL= (EBV x (pre Hct-post Hct))/(pre Hct).

*EBL is estimated blood loss during cesarean section.

### Sample size

The determination of the sample size was performed by comparing the quantity of intraoperative blood loss between women who had lower segment cesarean section delivery treated with intrauterine misoprostol and those who were given sublingual misoprostol, as this is the main outcome of our study. El-Sherbini et al. (2021) [4] reported that patients who were given intrauterine misoprostol experienced a mean ± SD of 692.39 ± 132.83 mL of intraoperative blood loss and that sublingual misoprostol may have clinical value in reducing blood loss by at least 10%. As mentioned, the least possible sample size was 96 women in each arm to be able to repudiate the null hypothesis via Student’s t test for independent samples with 90% power at an alpha level of 0.05. The calculation of the sample size was performed via G*Power software version 3.1.2 for MS Windows by Franz Faul at Kiel University in Germany.

### Statistical analysis

We reported the data in different ways—when convenient—as the means ± standard deviations, medians and ranges, percentages or frequencies (number of cases). The student’s t test was chosen for comparing numeric values among the study groups and ensuring independence. The chi-square (χ2) test was used to compare categorical data. An exact test was performed instead whenever the anticipated frequency was lower than five. Two-sided p values less than 0.05 were considered statistically significant. All the statistics were calculated via the SPSS computer program (Statistical Package for the Social Science, IBM Corp; Armonk, NY, USA) 22nd liberation for Microsoft Windows.

## Results

Regarding the demographic data of both the case and control groups, Table (1) shows that there were no significant differences in age, duration of pregnancy by date, ultrasound, parity, number of previous CSs or positive previous surgical history between the two groups. On the other hand, BMI and the number of previous abortions were significantly greater in the intrauterine misoprostol group than in the sublingual misoprostol group, but these findings were irrelevant to our study outcome.

**Table 1:** Demographic features of the study groups

	Intrauteine (*n* = 96)	Sublingual (*n* = 96)	*p* value
**Age (years)†**	**30.0 ± 5.3**	**28.5 ± 5.2**	**0.052**
**GA by date (Wks)†**	**39.2 ± 0.5**	**39.3 ± 0.6**	**0.116**
**GA by U/S (Wks)†**	**38.9 ± 0.4**	**39.0 ± 0.4**	**0.732**
**BMI (kg/m2)†**	**27.8 ± 1.3**	**27.0 ± 1.5**	< 0.001*
**Parity:‡**			
**Primipara**	**2 (2.1%)**	**0 (0.0%)**	**0.105**
**Para 1**	**27 (28.1%)**	**38 (39.6%)**	
**Para 2**	**67 (69.8%)**	**58 (60.4%)**	
**Number of previous CS:‡**			
**One**	**28 (29.2%)**	**38 (39.6%)**	**0.129**
**Two**	**68 (70.8%)**	**58 (60.4%)**	
**Number of abortions†**	**0.6 ± 0.9**	**1.1 ± 1.4**	0.002*
**Positive previous surgical history**	**39 (40.6%)**	**51 (53.1%)**	**0.083**

BMI: Body mass index; GA: Gestational age; †: Data are presented as mean ± SD; ‡: Data are presented as number of cases (%); *: Statistically significant difference (*p* < 0.05)

With respect to hemoglobin levels and hematocrit values preoperatively and postoperatively and the differences in Hb and HCT values, Table (2) reveals the absence of significant differences in the preoperative Hb and HCT values and the presence of statistically significant differences in the postoperative Hb and HCT values, and the differences in the Hb and HCT values were statistically lower in the intrauterine misoprostol group than in the sublingual misoprostol group.

**Table 2:** Comparison between mean value of preoperative, postoperative and difference in Hb& HCT between the study groups

	Intrauterine (*n* = 96)	Sublingual (*n* = 96)	*p* value
**Preoperative Hb (gm/dL)**	**11.3 ± 1.0**	**11.1 ± 1.0**	**0.068**
**Preoperative Hct%**	**34.5 ± 3.0**	**33.9 ± 3.1**	**0.183**
**Postoperative Hb (gm/dL)**	**10.5 ± 1.0**	**9.9 ± 1.1**	< 0.001*
**Postoperative Hct%**	**32.2 ± 3.2**	**30.8 ± 3.0**	0.002*
**Hb difference (gm/dL)**	**0.8 ± 0.2**	**1.2 ± 0.4**	< 0.001*
**Hct difference %**	**2.3 ± 0.6**	**3.1 ± 1.0**	< 0.001*

Data are presented as mean ± SD; *: Statistically significant difference (*p* < 0.05)

The intraoperative data displayed in Table (3) revealed an absence of significant differences between the two groups in terms of operative duration and content in the suction unit postoperatively. On the other hand, blood loss caused by soaked towels and EBL caused by the formula were significantly lower in the intrauterine misoprostol group than in the sublingual misoprostol group.

**Table 3:** Comparison of operative time, blood loss in soaked towels & blood loss in suction jar, mean value of EBL by the formula between the study groups

	Intrauterine (*n* = 96)	Sublingual (*n* = 96)	*p* value
**Time of operation (min.)**	**35.1 ± 5.8**	**36.0 ± 6.2**	**0.304**
**Blood loss by soaked towels (mL)**	**247.2 ± 60**	**327.2 ± 77**	< 0.001*
**Suction unit postoperative (mL)**	**177.6 ± 55**	**189.1 ± 82**	**0.258**
**EBL by the formula (mL)**	**417.4 ± 133**	**563.0 ± 196**	< 0.001*

EBL: Estimated blood loss; Data are presented as mean ± SD; *: Statistically significant difference (*p* < 0.05)

With respect to the postoperative data, there was no significant difference between the groups regarding the presence of too much blood loss of more than 1000 ml during the first six hours, the need for additional ecbolics, the demand for blood transfusion, the need for further surgical intervention for PPH and the presence of drug side effects, as shown in Table (4).

**Table 4:** Side effects occurrence in study groups, use of extra ecbolics, need for blood transfusion, need for additional surgical intervention (for PPH) and blood loss > 1000 ml

	Intrauterine (*n* = 96)	Sublingual (*n* = 96)	*p* value
**Excessive blood loss** **> 1000 ml in 1st 6 h**	**0 (0.0%)**	**2 (2.1%)**	**0.155**
**Need for Use of extra ecbolics**	**0 (0.0%)**	**2 (2.1%)**	**0.497**
**Need for blood transfusion**	**0 (0.0%)**	**0 (0.0%)**	
**Need for additional surgical intervention (for PPH)**	**0 (0.0%)**	**0 (0.0%)**	
**Shivering**	**21 (21.9%)**	**15 (15.6%)**	**0.267**
**Pyrexia**	**11 (11.5%)**	**19 (19.8%)**	**0.112**
**Nausea & Vomiting**	**5 (5.2%)**	**6 (6.3%)**	**0.756**
**Headache**	**13 (13.5%)**	**14 (14.6%)**	**0.836**

*Data are presented as number of cases (%)

## Discussion

Prevention of postpartum hemorrhage (PPH) and minimizing intraoperative blood loss are the main concerns of all obstetricians during cesarean delivery. It is well known that powerful myometrial contraction plays a fundamental role in controlling uterine bleeding through squeezing uterine vessels. Misoprostol possesses an adequate ecobolic effect that can be used for the management of postpartum hemorrhage [12]. Consequently, this study was conducted to validate the use of intrauterine misoprostol together with intravenous oxytocin infusion compared with sublingual misoprostol together with intravenous oxytocin infusion in reducing intraoperative blood loss and preventing postpartum hemorrhage during elective cesarean section.

Given these findings, we investigated 192 women and randomly divided them into two groups: the intrauterine misoprostol group, which included 96 women, and the sublingual misoprostol group, which included 96 women. We did not find a significant difference in maternal age, duration of pregnancy estimated by date or by ultrasound, parity, number of previous cesarean sections or positive previous surgical history between the two groups. On the other hand, BMI and the number of previous abortions were found to be significantly elevated in the sublingual group, but this was irrelevant to our study outcome.

Additionally, we observed no significant variation in the operative duration, which was 35.1 ± 5.8 min for the intrauterine group vs. 36.0 ± 6.2 min for the sublingual group, and no significant variation in the postoperative suction unit content, which was 177.6 ± 55 ml for the intrauterine group vs. 189.1 ± 82 ml for the sublingual group.

The primary outcome of our study was the amount of blood loss during and after cesarean delivery. Blood loss was lower in the intrauterine misoprostol group than in the sublingual group, with a statistically significant difference in the amount of blood loss observed between the soaked towel and EBL groups, as the amount of blood loss was 247.2 ± 60 ml in the intrauterine group compared with 327.2 ± 77 ml in the sublingual group and 417.4 ± 133 ml in the intrauterine group compared with 563.0 ± 196 ml in the sublingual group, with a p value < 0.001 for both. However, we did not find a significant difference between the groups in terms of extensive bleeding during the first six hours, the need for the use of additional ecbolics, the need for blood transfusion or the need for further surgical intervention.

We found a statistically significant difference in the postoperative Hb level between the two groups, with a value of 10.5 ± 1.0 gm/dl for the intrauterine group and 9.9 ± 1.1 gm/dl for the sublingual group, with a p value < 0.001. Additionally, the hematocrit value was significantly different: it was 32.2 ± 3.2 for the intrauterine group and 30.8 ± 3.0 for the sublingual group, with a p value of 0.002. Moreover, the difference in the Hb level and HCT value pre- and postoperatively revealed a significant disparity in favor of the intrauterine misoprostol group, as the difference in the Hb level was 0.8 ± 0.2 gm/dl for the intrauterine group and 1.2 ± 0.4 gm/dl for the sublingual group, with a p value < 0.001, and the difference in the hematocrit value was 2.3 ± 0.6 for the intrauterine group and 3.1 ± 1.0 for the sublingual group, with a p value < 0.001.

In our study, we used 400 mcg of misoprostol administered either intrauterine or sublingually. The adverse effects of misoprostol in our study were tolerated by most participants, with no significant difference between the groups. The most prevalent symptom was shivering, followed by pyrexia, whereas the least prevalent symptom was headache, followed by nausea and vomiting.

The effect of combining misoprostol with oxytocin was found to be related to the route of its administration, as evidenced by a network meta-analysis published by Gallos et al. 2019 [13]. In this context, in a comparative study examining the use of intrauterine versus sublingual misoprostol for preventing intraoperative and postpartum atonic bleeding in cesarean section, Mahrous et al. (2022) [12] used the standardized visual estimation technique to estimate the amount of blood loss during CS delivery and then calculated the volume of blood loss 6 h after delivery, which was similar to our method for assessing blood loss. These authors agreed with us and reported that 600 mcg of intrauterine misoprostol is beneficial for lowering the risk of PPH. In our trial, we employed 400 mcg of misoprostol in both the sublingual and intrauterine groups, and we observed the same effect but with a lower dose. Compared with sublingual misoprostol, they reported fewer blood transfusions, extra ecbolics, and subsequent interventions, as well as lower postoperative hemoglobin and hematocrit levels in the intrauterine misoprostol group. In addition, we agreed that there was no substantial difference in side effects between the groups.

Awoleke et al. [14] named sublingual misoprostol “sweet of life” when they conducted a comparative study to elaborate its superior effect on the prevention of postpartum hemorrhage over rectal use after vaginal delivery. They used 600 mcg of misoprostol to be given either rectally or sublingually and measured postpartum blood loss. In their study, sublingual use was associated with more shivering and fever, but it was more convenient for participants. On the other hand, Bagheri et al. (2022) [15] reported the superiority of rectal misoprostol over sublingual misoprostol in reducing cesarean section-related uterine bleeding when 200 mcg of misoprostol was used and demonstrated its effectiveness.

Another trial performed by El-Sherbini et al. 2021 [4] compared intraoperative intrauterine misoprostol administration with preoperative rectal misoprostol to minimize bleeding throughout and after cesarean birth. Assessment of blood loss was similar to that in our study, but they discovered that preoperative rectal insertion of misoprostol was just as effective as intrauterine misoprostol. However, intrauterine misoprostol was found to have better neonatal outcomes and to be more practical when given during cesarean delivery. They also documented no misoprostol-related adverse effects apart from postoperative shivering in either group.

When Alalfy et al. (2018) [1] investigated the effectiveness of intrauterine misoprostol during cesarean delivery for the prevention of primary postpartum hemorrhage, they compared intrauterine misoprostol together with oxytocin versus oxytocin alone and reported that combining intrauterine misoprostol (400 mcg) with oxytocin infusion was valuable for lowering postpartum blood loss and postpartum hemorrhage during elective CS delivery. They also reported that the intrauterine misoprostol group had considerably lower mean blood loss, as well as a greater decrease in hemoglobin and hematocrit levels, than did the oxytocin group. Furthermore, the intrauterine group required fewer blood transfusions, supplementary uterotonics or additional interventions. They also agreed with us that adverse events were comparable between groups.

Ghada et al. 2021 [5] also investigated intrauterine misoprostol but at a higher dose (800 mcg) simultaneously with oxytocin infusion versus oxytocin alone in reducing blood loss during cesarean section. They agreed with us and reported that the use of intrauterine misoprostol combined with oxytocin infusion in CS can significantly preserve hemoglobin and hematocrit and decrease the need for additional utertonics compared with oxytocin infusion alone, with no significant misoprostol-related side effects.

In a study by Gohar et al. [16], which investigated postpartum hemorrhage prevention with sublingual misoprostol versus oxytocin via different methods of blood loss assessment with a plastic blood collection drape placed beneath the patient before surgery, they reported that 400 mcg of sublingual misoprostol was more efficacious than 10 IU intramuscular oxytocin in lowering postdelivery blood loss, although the difference was statistically insignificant. On the other hand, they reported that the misoprostol group had significantly more side effects. In our opinion, although intramuscular oxytocin has prolonged action compared with intravenous injection, it has a delayed effect, which may explain their results. In our study, we combined misoprostol with IV oxytocin infusion to benefit from both immediate and long-lasting effects.

Lashin et al. (2022) [16] compared intrauterine misoprostol to intravenous oxytocin in preventing postpartum hemorrhage. They reported that the administration of oxytocin via intravenous infusion was more effective at lowering blood loss during CS than the administration of intrauterine misoprostol. They also reported statistically significant variation in blood loss among their study groups, with the intrauterine misoprostol group reporting more intraoperative, postoperative, and total blood loss than did the intravenous oxytocin group. Furthermore, they reported a noticeable discrepancy in pharmaceutical side effects, with shivering being more common among those taking intrauterine misoprostol and headache and vomiting being reported in those taking oxytocin. In our study, we combined both misoprostol and oxytocin in every patient while comparing the routes of administration, namely, the intrauterine and sublingual routes, and we detected no detectable differences in drug side effects.

A recent study by Caglar et al. (2023) investigated three regimens of the uterotonic agent oxytocin only versus carbetocin only versus a combination of oxytocin with intrauterine misoprostol and concluded that carbetocin is not superior to oxytocin alone or with intrauterine misoprostol in preventing postpartum hemorrhage. This finding was in line with our study [17]. 

According to the meta-analysis of Gallos et al. (2019) [13] investigating the effects of uterotonic drugs on the prevention and management of postpartum hemorrhage, adding misoprostol or ergometrine to oxytocin has a more favorable effect on postpartum hemorrhage prevention than does oxytocin alone but has more side effects. However, in their recommendation for prevention of postpartum hemorrhage in 2022, the International Federation of Gynecology and Obstetrics (FIGO) stated that the benefit of combining misoprostol or ergometrine with oxytocin outweighs the risk of adverse effects.

Eventually, excessive uterine bleeding during C section and the occurrence of postpartum hemorrhage can be safely avoided by adding misoprostol to the routine use of intravenous oxytocin infusion, which has mild or acceptable side effects. The limitation of our study is that it is a single-blind, not double-blind, randomized study.

## Conclusion

On the basis of our findings, we can infer that intrauterine misoprostol, when given with intravenous oxytocin infusion, is a reliable and effective way to reduce intraoperative blood loss and prevent postpartum hemorrhage in elective cesarean section and is more beneficial than sublingual misoprostol together with intravenous oxytocin infusion.

## Electronic supplementary material

Below is the link to the electronic supplementary material.

Supplementary Material 1

## Data Availability

The datasets used and/or analysed during the current study available from the corresponding author on reasonable request.
